# Stability of Direct Oral Anticoagulants Concentrations in Blood Samples for Accessibility Expansion of Chromogenic Assays

**DOI:** 10.3390/medicina59071339

**Published:** 2023-07-21

**Authors:** Anna Gavrilova, Jānis Meisters, Gustavs Latkovskis, Inga Urtāne

**Affiliations:** 1 Department of Pharmaceutical Chemistry, Faculty of Pharmacy, Riga Stradiņš University, LV-1007 Riga, Latvia; 2 Department of Pharmaceuticals, Red Cross Medical College of Riga Stradiņš University, LV-1009 Riga, Latvia; 3 Joint Laboratory, Pauls Stradiņš Clinical University Hospital, LV-1002 Riga, Latvia; 4 Latvian Center of Cardiology, Pauls Stradiņš Clinical University Hospital, LV-1002 Riga, Latvia; 5 Institute of Cardiology and Regenerative Medicine, University of Latvia, LV-1004 Riga, Latvia

**Keywords:** rivaroxaban, dabigatran, edoxaban, plasma, outpatient care, laboratory, temperature, storage, whole blood, aliquot, novel, tube

## Abstract

*Background and Objectives*: Direct oral anticoagulants (DOACs) are used for minimising the risk of thromboembolic events. In clinical practice, there is no need to measure DOAC concentration in the routine. Nevertheless, there are cases where such measurements are necessary, as the European Society of Cardiology’s guideline recommends. However, determining DOAC levels is not available for everyone due to chromogenic assay availability limitations from sample storage problems, as tests are performed only in a few healthcare settings. This study aimed to assess whether more applicable storage conditions could be used for transportation to provide chromogenic assays for outpatient healthcare and other hospitals’ practices. *Materials and Methods*: Chromogenic assays measuring anti-FXa (for rivaroxaban and edoxaban) and anti-FIIa (for dabigatran) were used. Concentrations were determined immediately after blood collection as baseline value: (1) after the storage of citrated whole blood in refrigerator (+2–8 °C); (2) of citrated plasma in refrigerator (+2–8 °C); and (3) of citrated frozen plasma (−20 °C) on the third and seventh days of storage. Acceptable change limits were considered stable if the deviation did not exceed ±20% of the baseline value. *Results*: The median (Cl 95%) baseline value of rivaroxaban was 168 (147–236) ng/mL; of dabigatran 139 (99–178) ng/mL; and of edoxaban—174 (135–259) ng/mL. The median deviation from a baseline value stored as citrate whole blood samples (+2–8 °C) was 5.4% and 3.4%; as citrated plasma (+2–8 °C) was 0.4% and −0.6%; and as citrated frozen plasma (−20 °C) was −0.2% and 0.2% on the third and seventh days of storage, respectively. *Conclusions*: Our data suggest that whole blood samples stored in a refrigerator, as well as citrated plasma samples stored in both the refrigerator and freezer, preserve DOAC concentration stable at +2–8 °C or −20 °C for up to 7 days, and are suitable for transportation, except for low-concentration samples.

## 1. Introduction

Direct oral anticoagulants (DOACs) like rivaroxaban, dabigatran, edoxaban, and apixaban are widely used in clinical practice. They are prescribed for stroke prevention in patients with atrial fibrillation, the treatment of venous thromboembolism (VTE), prophylaxis against VTE in high-risk surgical procedures, the prevention of recurrent thromboembolism, and the management of thrombosis in cancer patients [1,2,3,4]. DOACs offer advantages such as convenience (oral administration without routine monitoring), predictable pharmacokinetics, and a reduced potential for drug interactions, making them favourable options for anticoagulation therapy compared to vitamin K antagonists in these clinical conditions [4,5,6]. However, individual patient factors and specific clinical considerations should always be considered when determining the appropriate use of DOACs [4,7,8]. Furthermore, the consumption of DOACs is constantly growing due to their pharmacokinetic and pharmacodynamic properties [9].

There is still some uncertainty with regard to the clinical practice of monitoring DOAC therapy in patients with safety issues. In compliance with the use of DOAC guidelines, extra caution should be taken in patients with a bleeding history, renal failure, hepatic impairment, and drug therapy interactions [10,11,12,13]. The goal of drug therapy is to be safe and effective, but it has already been observed that the risk of bleeding increases in patients with higher blood levels of DOACs [14]. Regular DOAC laboratory monitoring is not considered necessary, but measuring anticoagulant activity using quantitative methods can be beneficial. Firstly, it allows for assessing therapeutic drug levels in patients receiving anticoagulant therapy to prevent thromboembolic conditions. Monitoring DOAC concentrations helps clinicians to ensure patients are within the therapeutic range, minimising the risk of bleeding or thrombotic events. Secondly, measuring DOAC concentrations is crucial in situations that require dose adjustment. In cases of suspected drug interactions, renal impairment, or bleeding complications, knowing the DOAC concentration in a patient’s blood can aid in making informed decisions regarding dosage modifications or treatment interventions. Furthermore, quantifying DOAC concentrations is vital in emergencies, such as trauma or urgent surgeries, where the reversal of anticoagulant effects may be necessary. Lastly, DOAC concentration measurements provide valuable data for pharmacokinetic and pharmacodynamic studies. They contribute to a better understanding of drug metabolism, elimination, and individual variability, aiding in developing personalised medicine approaches [4,5,15].

Several factors need to be considered to determine the most appropriate laboratory test for monitoring DOAC levels. These include the availability of the test, the extent of information required (qualitative or quantitative assay), and the time it takes to receive the test results [16].

Standard coagulation assays such as prothrombin time (PT), activated partial thromboplastin time (aPTT), and thrombin time (TT) have been studied. The widespread availability of these methods in all clinical laboratories provides a significant advantage, particularly in emergencies, as they effectively support clinical decision-making processes. However, assays such as the PT, aPTT, and TT are unpredictable in assessing DOAC activity due to a poor correlation with DOAC plasma concentration. It is important to note that there can be variability in the results obtained using different reagents, as the sensitivity of the reagent to the specific DOAC may differ [16,17,18,19,20,21].

Thus, the ‘gold standard’ for determining the plasma levels of DOAC is liquid chromatography-tandem mass spectrometry (LC-MS/MS). LC-MS/MS is an advanced analytical method used to measure plasma concentrations of all DOACs [22,23,24,25]. This technique involves detecting the drug molecules in a pretreated plasma sample. Unlike other methods, LC-MS/MS directly quantifies the drug concentrations and does not require calibration using a known concentration sample. The routine use of LC-MS/MS for determining the concentration of DOACs is considered impractical in routine clinical care practice due to factors such as high equipment costs, the requirement for qualified personnel, and the extended assay time.

In previous studies, chromogenic assays were highly correlated with LC-MS/MS methods, so these can be used as a reliable and efficient alternative to LC-MS/MS to quantify DOAC concentration rapidly [16,26]. The BIOPHEN^®^ DiXaI and INNOVANCE^®^ DTI assays are specific chromogenic assays used in laboratory settings to measure anticoagulants. These chromogenic assays offer a rapid quantifying concentration of DOACs. Both assays provide quantitative results, allowing healthcare providers to determine the drug concentration of the anticoagulant therapy and ensure that patients receive the appropriate dosage or the needed dose adjustment [16,26,27,28].

However, high-accuracy methods have limited accessibility for patients and clinicians. In Latvia, LC-MS/MS is primarily utilised for scientific purposes, while chromogenic assays are exclusively available for inpatients in clinical university hospitals [19,27,28,29]. In emergency department settings, assessing acute patients frequently necessitates the utilisation of chromogenic assays. It allows for differentiating between patients with an over- or underdose of prescribed therapy once the acute event has happened.

It is important for patients to have regular follow-up visits and adhere to their prescribed treatment regimen to ensure optimal outcomes and minimise the risk of complications. For ambulatory practice clinicians, there is difficulty in considering anticoagulation therapy safety because chromogenic assays are unavailable, despite the European Society of Cardiology’s recommendations to measure DOAC concentration in complicated cases [5]. Yet, the storage provided by manufacturers for transporting primary tubes in no more than 4 h at room temperature or separated plasma in −18 °C conditions is impractical for transporting blood samples, especially from distant areas. Ensuring access for all specialists to measure DOAC levels for patients is imperative. This study’s approach will facilitate the monitoring and optimisation of DOAC therapy by utilising chromogenic assays. This will lead to improved care and a reduced risk of adverse events for a larger patient population. For this reason, we considered studying the stability of DOAC in blood samples for the accessibility expansion of chromogenic assays [19,27,28,29].

This study aimed to assess whether DOACs containing blood sample storage conditions (storage durations and temperatures) are more applicable for transportation to provide anti-FXa and anti-FIIa chromogenic assays for outpatient healthcare and other hospitals’ practice.

## 2. Materials and Methods

### 2.1. Study Design

This was an in vitro study performed at Pauls Stradiņš Clinical University Hospital (PSCUH), Riga, Latvia, from August to December 2022. Ethical approval for this study was obtained from the Central Medical Ethics committee of Latvia on 4 November 2021 (No. 10900) and carried out in accordance with the Declaration of Helsinki.

This study involved patients scheduled for planned outpatient electrical cardioversion as part of their hospital visit. Blood samples were collected specifically for this study after informed consent was obtained. The inclusion criteria were: (1) ≥18 years and (2) receiving rivaroxaban, dabigatran or edoxaban therapy. Meanwhile, the exclusion criteria were: (1) anaemia and (2) the inability to obtain the required amount of blood. Samples with no detectable baseline concentrations, e.g., below LoD or exceeding calibration reference curve (for Xa inhibitors out of 5–500 ng/mL and for IIa inhibitors out of 20–500 ng/mL) were excluded from further analysis. 

### 2.2. Sample Collection and Storage Conditions

One sample consisting of two sodium citrate 3.2% 2.7 mL tubes (BD Vacutainer^®^) was taken from each patient. All blood tubes were filled with blood to the required volume indicated by the black mark on each tube. After collecting blood samples, a department employee transported them to the laboratory within 4 h at room temperature from sampling time. Afterwards, DOAC concentration determination analysis was conducted immediately.

**Figure 1:**
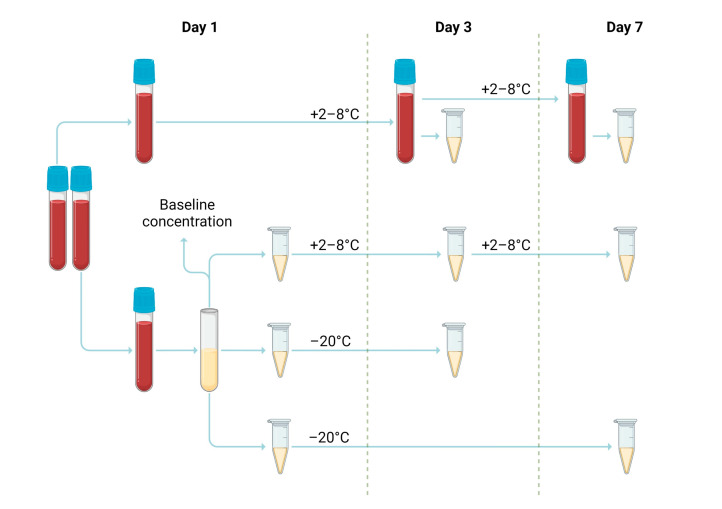
Collected samples’ processing, storage duration, and conditions for the determination of in vitro stability.

DOAC concentrations were measured by chromogenic assays, which are described in more detail in Section 2.3. The first tube was centrifugated (2500× *g* for 15 min) and tested to determine DOAC baseline value. The rest of the plasma was separated into three aliquots—one for storage at +2–8 °C and two were frozen below −20 °C. The second tube was left unprocessed (whole blood) and stored in a refrigerator (+2–8 °C). The repeated assay was performed for each temperature regime on the third and seventh days of storage. Before analysis, whole blood sample was centrifugated and plasma was used. The rest of the whole blood was gently turned upside down three to four times and returned to the refrigerator (+2–8 °C) for further storage. Plasma samples were directly used for analysis without any prior processing or additional preparation. Each aliquot was measured three times. Figure 1 demonstrates the storage duration and conditions of DOAC samples’ (whole blood and plasma) in vitro stability.

### 2.3. Chromogenic Assays

To assure quality and trueness of measurements, two levels of quality controls were measured in a day of testing that were evaluated following the quality assurance procedure of Joint Laboratory PSCUH. Methods were validated by Siemens and verified in the Joint Laboratory of PSCUH using patient samples, external quality control material, and internal quality control data. CV obtained during verification at low and high concentrations were as follows: dabigatran, 9.1% and 4.1%; rivaroxaban, 9.0% and 7.1%; edoxaban, 4.9% and 2.7%. External quality control is performed yearly and was performed throughout the research.

#### 2.3.1. Anti-Factor Xa (Fxa) Assay

For the determination of rivaroxaban and edoxaban “Siemens “anti-Xa” calibrated with DOAC-specific calibrator “BIOPHEN^®^ DiXaI” reagent (Hyphen Biomed, Neuville-sur-Oise, France) was used, which is an automated chromogenic assay set up on Sysmex CS-2500 haemostasis analyser. The reagent contained factor Xa and chromogenic substrate specific for Xa. In the presence of direct factor Xa inhibitors, factor Xa was inhibited and the chromogenic substrate remains uncleaved. The measured signal was then compared to inhibitor-specific reference curve, allowing for the quantification of the inhibitor concentration in the sample. The calibration curve of rivaroxaban was set up using commercial standards from Siemens, but for edoxaban, using reference material from the manufacturer Biophen.

#### 2.3.2. Anti-Factor Iia (FIIa) Assay

Dabigatran concentration in plasma was measured by commercial competitive chromogenic assays (INNOVANCE^®^ DTI Assay, Siemens Healthineers, Erlangen, Germany) using haemostasis analyser Sysmex CS-2500 (Siemens). To measure the concentration, Direct Thrombin Inhibitor assay (Siemens) was used. The reagent contained bovine thrombin and thrombin-specific chromogenic substrate. By adding excess thrombin to citrated plasma that contained a direct thrombin inhibitor, the portion of the thrombin was inactivated. The remaining portion of uninhibited thrombin then cleaved a specific chromogenic substrate, causing the release of dye. The rate of the substrate cleavage was determined by the increase in absorbance at 405 nm wavelength. The obtained result was then compared against the calibration curve that was defined using commercial Dabigatran Standarts (Siemens).

### 2.4. Statistical Analysis

Statistical Package for the Social Sciences (IBM SPSS Statistics 29.0) and GraphPad Prism 9.0 were used for data analyses. Non-parametric methods were employed because the data obtained did not correspond to a normal distribution. Descriptive statistics are presented as median and 95% confidence level (Cl). For group comparison, Levene’s test was applied. All of the statistical tests were two-sided using a significance level of 0.05. Acceptable stability was defined as 80–120% of the baseline result, and any deviation greater than 20% was considered clinically significant [30,31,32].

## 3. Results

A total of 68 DOAC-containing samples were collected, of which four were excluded from stability determination. The baseline values of three samples were out of the detectable concentration range, and one sample was haemolysed before testing. As a result, 32 rivaroxaban, 11 dabigatran, and 25 edoxaban samples were analysed. The median (Cl 95%) baseline value of rivaroxaban was 168 (147–236) ng/mL; of dabigatran, 139 (99–178) ng/mL; and of edoxaban, 174 (135–259) ng/mL. Table 1 provides an overview of the stability results. The median deviation from a baseline value stored as citrate whole blood samples (+2–8 °C) was 5.4% and 3.4%; citrated plasma (+2–8 °C) was 0.4% and – 0.6%; and citrated frozen plasma (−20 °C) was – 0.2% and 0.2% on the third and seventh days of storage, respectively. Using Levene’s test, there was no significant variance between baseline measurements and the results were obtained on the third and seventh days (*p*-value > 0.05).

### 3.1. Stability of DOACs in Whole Blood at +2–8 °C

**Figure 2:**
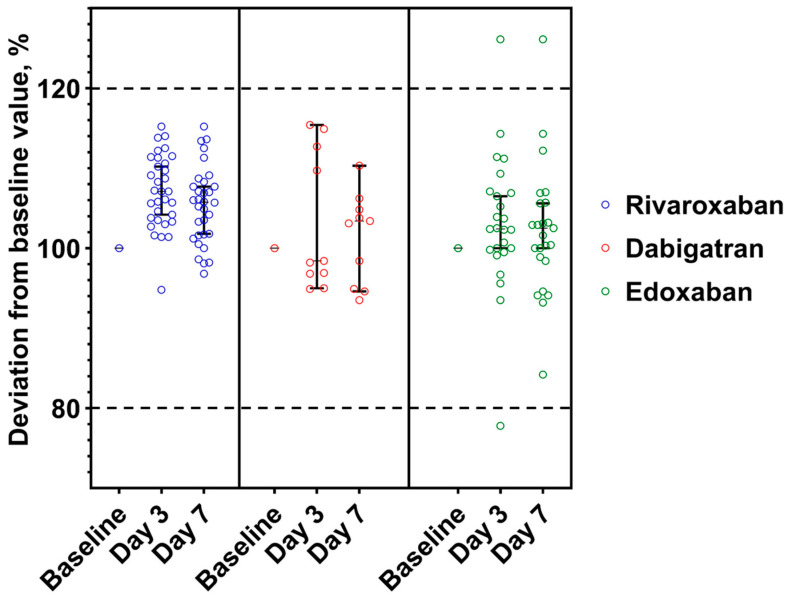
DOAC concentrations deviations from baseline value expressed as a percentage and confidence level 95% in the whole blood samples stored at +2–8 °C. Range of acceptance is indicated by dotted lines. The median concentration, expressed as a percentage of the baseline concentration, and the 95% confidence interval are shown.

The stability data of samples stored as unprocessed whole blood at +2–8 °C is presented in Figure 2. One dabigatran and two edoxaban and samples exceeded ±>20% deviation on the third day. More detailed data are available in Table 2.

### 3.2. Stability of DOACs in Plasma at +2–8 °C

**Figure 3:**
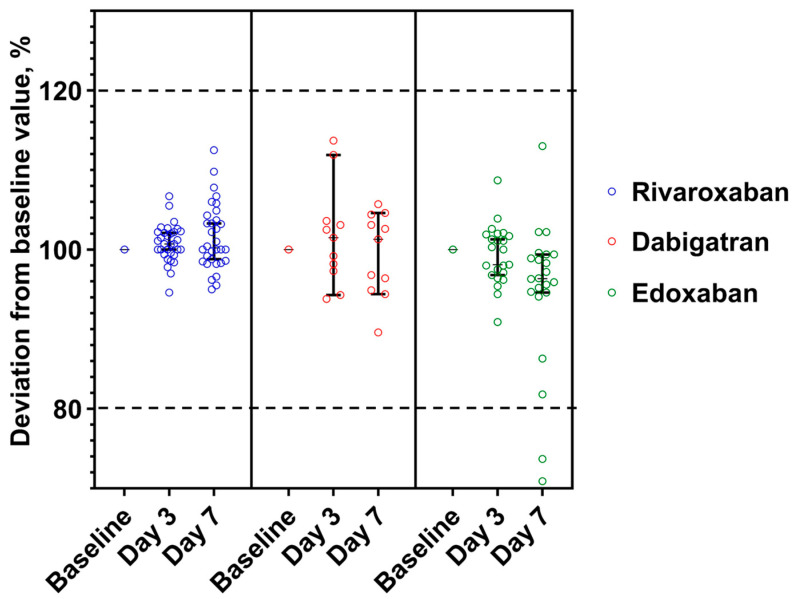
DOAC concentrations deviations from baseline value expressed as a percentage and confidence level 95% in the citrated plasma stored at +2–8 °C. Range of acceptance is indicated by dotted lines. The median concentration, expressed as a percentage of the baseline concentration, and the 95% confidence interval are shown.

None of the rivaroxaban and dabigatran aliquots showed a significant deviation (>±20%) from the baseline value (Figure 3). Nevertheless, two edoxaban samples (baseline values 9 and 19 ng/mL) had −31.6% and −33.3% on the third day and, consequently, −26.3% and −22.2% on the seventh day.

### 3.3. Stability of DOACs in Frozen Plasma at −20 °C

**Figure 4:**
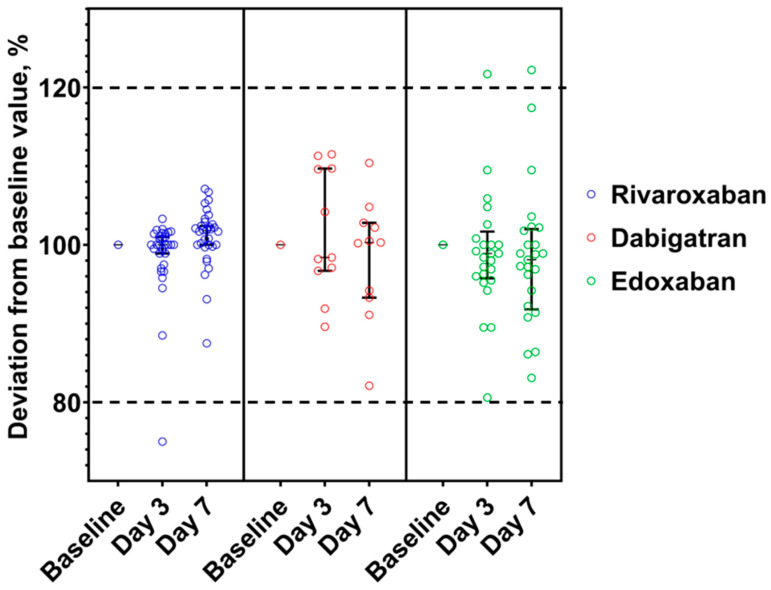
DOAC concentrations deviations from baseline value expressed as a percentage and confidence level 95% in the citrated plasma stored at −20 °C. Range of acceptance is indicated by dotted lines. The median concentration, expressed as a percentage of the baseline concentration, and the 95% confidence interval are shown.

Stability results of DOAC in frozen plasma are shown in Figure 4. Neither dabigatran samples showed a significant deviation (>±20%) from the baseline value, unlike one rivaroxaban and two edoxaban aliquots (Table 3).

## 4. Discussion

The key findings of this study indicate that samples generally did not deviate significantly outside of ±20% under the two studied conditions as alternatives to the standard requirements of centrifugated plasma. Notably, the stability of whole blood samples stored in a refrigerator at +2–8 °C on the third and seventh days was also observed throughout the study. Even though all temperature regimes did not significantly impact concentrations in citrated blood or citrated plasma within 7 days, transporting samples in a refrigerator at +2–8 °C is as desirable as the ones frozen at −20 °C. Obtaining the optimal storage conditions for the stability of DOAC concentrations in blood samples enhances diagnostic availability, improving patient care within routine clinical practices.

The current laboratory practice is to transport sodium citrate tube samples from departments and other locations at room temperature as fast as possible, separating them from other tubes transported in 2–8 °C conditions. Our results also differ from manufacturer recommendations that dictate that the transportation should be performed at room temperature in 4 h, or if not possible, plasma should then be separated right after sampling and freezing. Our results show that there is no need to use centrifugation for plasma separation right after collection. Instead, the samples can be centrifuged shortly before analysis because whole blood samples also showed stability within 7 days.

Determining an acceptable variation coefficient presents a significant challenge and a potential area for future investigation. One way to conduct this is to look at biological variation data. Nonetheless, studies that are directed to determine intra- and inter-individual variation for DOACs are lacking, as is common for almost all drugs. Based on the study by Reda et al., inter-individual biological variations for various DOACs exceed 40% at peak and 60% at trough concentrations and intra-individual CV above 20%, so the acceptable CV was set at 20% even though the analytical CV was below 10% [30,31,33]. It is essential to take into consideration that the lower concentrations had higher deviations from baseline values in percentages, but clinically, this was not significant, e.g., the sample’s baseline value was 24 ng/mL with 37.5% deviations on the third day, which meant 33 ng/mL (Table 2 and Table 3).

The pharmacokinetic data of DOACs provide evidence supporting their wide therapeutic range [16]. Despite significant advancements in DOACs as anticoagulant medications, there is still limited knowledge regarding the precise relationship between DOAC plasma concentration and the anticoagulation effect. While anticoagulants like warfarin have well-established therapeutic monitoring protocols based on international normalised ratio (INR) values, DOACs are generally administered at fixed doses without the routine monitoring of drug levels. Nonetheless, anti-FXa and anti-FIIa assays can be valuable in evaluating therapy safety because it was already observed that patients with higher anticoagulant levels had higher bleeding risks [14]. Also, polypharmacy’s and drug–drug interactions’ impact on anticoagulation therapy can be estimated by DOAC level quantification. More studies are needed to determine the exact DOACs’ therapeutic ranges for avoiding haemorrhages and providing sufficient thromboembolic prophylaxis.

Chromogenic assays of DOACs are not helpful in measuring patients’ long-term adherence levels through drug concentration in plasma. For instance, the elimination of rivaroxaban from plasma occurs with terminal half-lives of 5 to 9 h in young individuals and with terminal half-lives of 11 to 13 h in older people. Expected plasma trough levels in patients treated for atrial fibrillation are 12–137 ng/mL [5,10]. In comparison, the chromogenic assay limit of quantitation is 5 ng/mL or higher, depending on the used reagents [16]. Consequently, lower values will not be identified, or obtained results could not confirm that the patient used medicine for at least one week without interruption.

The strength of this study was its ability to offer solutions for chromogenic assays to be utilised by a wider range of patients and clinicians beyond those in leading hospitals. This study provides verified information on optimal blood sample storage conditions for transportation from peripheral locations. In more distant healthcare facilities, where specific equipment may be limited, this information is needed to ensure the stability of samples during transit. These results can promote a more common usage of chromogenic assays in clinical practice to provide the effective and safe utilisation of DOACs. 

One of the limitations of this study was the decision not to investigate the stability of samples containing apixaban because of the lack of patients taking it. Similarly, we had few dabigatran samples due to non-wide use in practice. Additionally, budget limitations prevented us from having a larger number of samples. A small sample size increases the risk of type II errors, which could potentially affect the power and reliability of the study’s findings. It would be highly beneficial to replicate and validate these results in research with a more substantial sample size. Ethical approval protocol limitations to use only two blood tubes of a sample from one patient and only 2.7 mL of sodium citrate 3.2% tube availability in stock did not allow us to have two unprocessed whole blood tubes to be stored in refrigeration instead of one, thus avoiding sample reuse on the third day.

## 5. Conclusions

Our data suggest that two more convenient alternatives, whole blood samples stored in a refrigerator, as well as citrated plasma samples stored in both the refrigerator and freezer, preserve DOAC concentration stable at +2–8 °C or −20 °C for up to 7 days, except for the need to precisely determine low concentrations of DOAC. These storage conditions are suitable and convenient for DOAC-containing blood samples transportation from other healthcare points to perform chromogenic assays.

## Figures and Tables

**Table 1:** Summary of concentrations at specific time points under different sample storage conditions.

DOAC	Sample (Temperature)	Median (Cl 95%), ng/mL (Median Deviation from Baseline Value, %)				
		Baseline Value	Day 3	*p*-Value *	Day 7	*p*-Value *
Rivaroxaban	Whole blood (+2–8 °C)	168 (147–236)	187 (157–249) (7.1)	0.865	182 (154–245) (5.7)	0.963
	Plasma (+2–8 °C)		173 (148–238) (0.7)	0.997	171 (149–238) (0.2)	0.993
	Plasma (−20 °C)		168 (146–235) (0.0)	0.977	173 (149–239) (1.8)	0.972
Dabigatran	Whole blood (+2–8 °C)	139 (99–178)	133 (103–185) (−1.6)	0.985	130 (100–184) (3.4)	0.882
	Plasma (+2–8 °C)		137 (102–179) (1.5)	0.874	134 (98–181) (1.3)	0.865
	Plasma (−20 °C)		126 (99–183) (−1.6)	0.993	118 (95–176) (0.3)	0.906
Edoxaban	Whole blood (+2–8 °C)	174 (135–259)	178 (139–264) (2.4)	0.949	179 (138–261) (2.5)	0.990
	Plasma (+2–8 °C)		169 (134–257) (−1.9)	0.984	180 (125–255) (−3.7)	0.962
	Plasma (−20 °C)		167 (133–254) (−1.1)	0.967	159 (130–251) (−1.9)	0.869

* The *p*-value was calculated based on Levene’s test, comparing the median values between baseline measurements and the results obtained on the analysis day.

**Table 2:** The concentrations of the samples exceeded deviations of more than ±20% stored as citrated whole blood samples at a temperature +2–8 °C.

DOAC	Concentration, ng/mL (Deviation from Baseline, %)		
	Baseline Value	Day 3	Day 7
Dabigatran	24	33 (37.5)	32 (33.3)
Edoxaban	9	7 (−22.2)	14 (55.6)
Edoxaban	23	29 (26.1)	29 (26.1)

**Table 3:** The concentrations of the samples exceeded deviations of more than ± 20% stored as citrated plasma at a temperature of −20 °C.

DOAC	Concentration, ng/mL (Deviation from Baseline, %)		
	Baseline Value	Day 3	Day 7
Rivaroxaban	8	6 (−25.0)	7 (−12.5)
Edoxaban	9	14 (55.6)	11 (22.2)
Edoxaban	23	28 (21.7)	27 (17.4)

## Data Availability

Not applicable.
